# Increased Complexities in Visual Search Behavior in Skilled Players for a Self-Paced Aiming Task

**DOI:** 10.3389/fpsyg.2017.00987

**Published:** 2017-06-14

**Authors:** Jingyi S. Chia, Stephen F. Burns, Laura A. Barrett, Jia Y. Chow

**Affiliations:** ^1^Physical Education and Sports Science, Nanyang Technological UniversitySingapore, Singapore; ^2^School of Sport, Exercise and Health Sciences, Loughborough UniversityLoughborough, United Kingdom; ^3^Institute for Sports Research, Nanyang Technological UniversitySingapore, Singapore

**Keywords:** badminton serve, visual behavior, expertise, quiet eye, eye-tracking, deception

## Abstract

The badminton serve is an important shot for winning a rally in a match. It combines good technique with the ability to accurately integrate visual information from the shuttle, racket, opponent, and intended landing point. Despite its importance and repercussive nature, to date no study has looked at the visual search behaviors during badminton service in the singles discipline. Unlike anticipatory tasks (e.g., shot returns), the serve presents an opportunity to explore the role of visual search behaviors in movement control for self-paced tasks. Accordingly, this study examined skill-related differences in visual behavior during the badminton singles serve. Skilled (*n* = 12) and less skilled (*n* = 12) participants performed 30 serves to a live opponent, while real-time eye movements were captured using a mobile gaze registration system. Frame-by-frame analyses of 662 serves were made and the skilled players took a longer preparatory time before serving. Visual behavior of the skilled players was characterized by significantly greater number of fixations on more areas of interest per trial than the less skilled. In addition, the skilled players spent a significantly longer time fixating on the court and net, whereas the less skilled players found the shuttle to be more informative. Quiet eye (QE) duration (indicative of superior sports performance) however, did not differ significantly between groups which has implications on the perceived importance of QE in the badminton serve. Moreover, while visual behavior differed by skill level, considerable individual differences were also observed especially within the skilled players. This augments the need for not just group-level analyses, but individualized analysis for a more accurate representation of visual behavior. Findings from this study thus provide an insight to the possible visual search strategies as players serve in net-barrier games. Moreover, this study highlighted an important aspect of badminton relating to deception and the implications of interpreting visual behavior of players.

## Introduction

Our eye movements typically occur in a top–down manner whereby we look at wherever the task at hand requires us to Chen and Zelinsky (2006). Likewise, in sports, appropriate visual attention precedes and determines effective movement behavior (Savelsbergh et al., 2002) and the ability to pick up relevant visual information has been highlighted as a discriminating factor of superior performances (Williams et al., 2004; Mann et al., 2007; Broadbent et al., 2014). The process of selecting and attending to visual information is not random but based on deliberate visual search strategies (Memmert et al., 2009). There exists evidence of higher skilled players exhibiting a visual pattern made up of fewer fixations of longer durations compared with their less skilled counterparts in several sports such as soccer, volleyball, basketball, and shooting (Vickers, 1996; Lee et al., 2009; Cañal-Bruland et al., 2011; Piras et al., 2014).

Quiet eye (QE) is the final fixation point during the preparatory phase of a goal-directed movement (Vickers, 1996). QE research has been conducted in various sporting tasks and it has been shown to be characteristic of visual behavior especially in self-paced aiming tasks such as golf, archery and shooting (Mann et al., 2007). In such tasks, access to pertinent visual stimuli and the ability to process them effectively whilst maintaining an optimal level of concentration are essential for successful performances (Causer et al., 2010). While QE duration differs depending on the specific task demands, there exists robust literature for QE effects both on an inter-individual level with higher skilled players exhibiting longer QE durations than the less skilled (Lee et al., 2009; Causer et al., 2010) as well as on an intra-individual level where longer QE durations were associated with successful outcomes (Williams et al., 2002; Wilson and Pearcy, 2009). It has been suggested that a longer QE ensures both the pre-planning of movement (Mann et al., 2011) and its subsequent online detection and use of visual information for guidance (Oudejans et al., 2002; Vine et al., 2013). However, despite the robust support for QE as a key characteristic of skilled performance, little has been done on racket games, leaving an important gap in the research literature. Especially for the case of the serve task, the individual has ample time to aim before executing the serve. Therefore, there is a possibility that this concept of QE can be extended to service in racket games, providing a valuable method of analyzing the perceptual-cognitive skills involved.

One example is badminton. The badminton serve is an important shot with the dual aims of minimizing the opponent’s chance of attacking and increasing one’s chances of eventually winning the rally (Downey, 1982; Hussain et al., 2011; Seth, 2016). This is normally done by sending the opponent out of position, away from the center of his court, thereby creating space for an attacking shot. Moreover, the recent change in scoring system states that a player can score a point regardless of whether he started the rally; placing higher weight on the serve itself (Badminton World Federation, n.d.). Given the dynamic nature of the game, every shot taken alters the present situation and creates a new one which will either be in one player’s advantage or none; a sort of status quo situation (Downey, 1982).

In the singles discipline, there are two main serves – the short and the long serve, each with its pros and cons (Mack, 2016). It has been suggested that the long serve is the more popular of the two; sending the shuttle right to the back of the court automatically gives the server more time to take up position and hence even if he does not win the point outright, this will put him in a good position to win the next. However, in an analysis on 11 elite singles players over 10 matches, Tong and Hong (2000) found virtually the same number of short and long serves used. The choice of serve depends on several factors, but not limited to, the opponent’s position, playing style as well as the intention of the server. For instance, in situations where the opponent stands too far out in anticipation of a serve to the back of the court, using a short serve here will often put him in difficulty (Mack, 2016). It is thus important that one is never too quick to serve but looks at the opponent, noting especially the standing position before deciding which serve to use to one’s advantage. Mixing the serve types will also keep the opponent guessing rather than taking up a stance near the base line with impunity if one constantly serves long (Dick, 2011).

As such, players must be able to accurately utilize the correct parameters and informational constraints; in this case, integrating visual information from the shuttle, racket, target, and opponent (Wilson et al., 2015). A player can win a point if the opponent is unable to return the serve successfully. Therefore, apart from aiming to get the shuttle to their desired spot, it is advantageous for players to manipulate or minimize the perceptual information they present to the opponent, making it hard for them to anticipate the serve type. It can thus be said that the presence of an opponent may alter one’s visual strategy. When performing the long serve, less skilled players have the tendency of displaying a big swing action. This, however, is not necessary as the wrist alone can send the shuttle to the back of the court in a long serve (Mack, 2016). The actions for performing a short or long serve should be as similar as possible to keep the opponent off balance, preventing him from attacking on the serve (Grice, 2007). The serve, therefore, provides a unique task for the examination of visual behavior.

To date, there exists no information on the visual strategy adopted during the serve. However, two classic studies by Abernethy and Russell (1987a,b) examined the visual search patterns of players in a serve anticipation task. Higher skilled players were found, to demonstrate superior ability in picking up more relevant informational cues at an earlier time stage (e.g., opponent’s playing side arm) vs. relying entirely on the opponent’s racket action to anticipate serve direction. However, unlike other sports, visual search characteristics of both groups of players were found to be similar, highlighting “cue” usage differences rather than specific visual search patterns differences. Likewise, a more recent study by Alder et al. (2014) reported no differences in the number of fixations as a function of skill during anticipation of badminton shots. Skilled players, however, had longer fixation durations compared to the less skilled.

It is worth noting the distinction between the visual information garnered for an interceptive or anticipatory task such as returning a serve and that for a self-paced task (e.g., serving) (Vine and Klostermann, 2016). A strong temporal constraint is present in an interceptive or anticipatory task as the visual information is available at very specific moments of the opponent’s action (Paterson et al., 2013). Theoretically, from a more cognitive approach to understanding visual perception, this demands that the individual extracts from the most valuable sources of visual information based on their semantic knowledge to quickly initiate a motor response (Shim et al., 2005). Here, successful performance is an outcome of effective information processing with higher skilled individuals directing their visual attention to relevant cues based on their knowledge, often within the very first eye movement (Castelhano and Henderson, 2007). From an ecological theory of perception and action (Gibson, 1979), the role of direct perception guiding action has been emphasized and provides a dynamic and representative explanation of how movement is controlled where information from the environment continually guides actions. The interaction between the “actor” (player) and the environment is critical in the provision of perceptual information in guiding action. Exploratory behaviors during practice allows affordances (functional movement possibilities) to be acquired. Perceptual information is a relation between the variables in the ambient arrays (in the environment) and the perceiver (Withagen and van der Kamp, 2010). As such, it cannot be assumed that similar visual behavior would be observed between returning a serve and serving. The lack of a temporal constraint during service, coupled with the need to maximize information extraction yet minimize cues presented to their opponent, may thus see higher skilled players taking a longer preparatory time to serve and exhibiting more fixations than the less skilled. Conversely, the less skilled may have fewer sources of useful information available and hence, are likely to display fewer fixations.

This warrants the need to identify the visual patterns that players undertake when serving which can provide valuable insights to the visual search behaviors and its role in movement control for such self-paced tasks. Therefore, the aim of the present study was to examine skill-related differences in visual behavior during the badminton serve. The comparison of strategies used by players of different skill levels would provide an insight to skill-related differences as well as any individual variations in visual behavior. Findings may also provide insights on any cue manipulation by servers. Given the temporal nature of the badminton serve and expertise of players, we hypothesized that higher skilled players would exhibit greater number of fixations on more areas of interest. Secondly, while little is known about whether QE is relevant in these situations, we know that QE is a variable that has been shown to reliably distinguish expertise. As such, it is likely that QE duration would be longer for the skilled player as compared to the less skilled. Finally, considering the greater number of fixations and longer QE duration, we hypothesized that the skilled players would take a longer preparatory time prior to serving.

## Materials and Methods

### Participants

A total of 24 players participated in the present study. They were 12 (six males and six females) skilled players (age: 22.4 ± 1.38 years) and 12 (six males and six females) less skilled players (age: 25.5 ± 3.45 years). The skilled players have had at least 3 years of competitive experience (9.25 ± 3.25 years) at college (*n* = 6) and national level (*n* = 6). They trained at least twice a week with each session lasting at least 2 h. The less skilled players had no competitive experience, played badminton recreationally and had undergone no formal badminton training. In addition, a skilled badminton player (age: 22 years) with 8 years of competitive experience at college level was recruited to act as the opponent (to receive serve) for all players in this study. Prior to the data collection, this skilled player was briefed and familiarized with the task requirements to ensure consistency and accuracy in the execution of the task, as described in the following paragraph. The study was approved by an institutional review board and adhered to the guidelines for ethical practice. Participation was voluntary and informed consent was received from each participant.

### Task

Participants were required to perform 30 serves from anywhere in the right service court to the skilled live opponent diagonally across court, as one would do during a singles match situation. To maintain the representativeness of the task, participants were not restricted on the type of serve – height, length or pace of serve – used. The participant was also required to return the shot from the opponent who returned the service, mimicking a rally. Ensuring representativeness is critical as it emphasizes the need to ensure that experimental task constraints represent the task constraints of the performance or training/learning environment (Pinder et al., 2011). This will then allow the performer to demonstrate the perceptual and movement behaviors that actually occurs in real game conditions (thus, there were no restrictions to serve types).

### Apparatus

The experiment was conducted on an indoor air-conditioned badminton court. Participants were allowed to use their own badminton rackets or those without were provided one. Feathered shuttles (Yonex Aerosensa-50) were used and replaced regularly throughout the duration of the study. Eye movement data were collected using an Applied Science Laboratories (ASL) Mobile Eye-XG movement system, a video-based monocular corneal-reflection system which measures point of gaze (at 30 Hz) based on the vertical and horizontal distances between the center of the pupil and corneal reflection after correcting for second-order effects. The system is accurate to within 0.5 to 1° of visual angle with a visual range of 60° horizontal and 40° vertical. In addition, a side-on digital video camera (Casio Exilim EX-FH 100) was used to determine the point of racket-shuttle contact (at 30 Hz).

### Procedure

Upon arrival at the indoor court and after briefing by the researcher, participants were fitted with the eye tracker. Following a nine-point grid calibration at the side of the court, they were given 10 min to rally with the skilled opponent as a warm-up and for familiarization purposes. The distance between the participant and the calibration grid was ∼3 m, similar to that between the participant and the net during service. Before each trial, the participants were instructed to walk to wherever they wished to serve from (within the right-side service box) and then look toward the tester with a clapperboard to signal the start. This also served as a calibration check and if necessary, line of gaze was re-calibrated before resuming the test. Participants were tested individually and they were allowed to rest if necessary. Each session lasted about an hour.

### Measures

To examine visual behavior, the following measures were obtained and values recorded were averaged across all trials for each participant.

#### Trial Length

This was the preparatory time taken prior to serving for each trial. Each trial was tracked from the first frame demarcating the start signal (see Procedure) up to the instance where the shuttle was served (racket-shuttle contact).

#### Fixation Count and Location

A fixation was defined as the time when the individual’s gaze remained stationary at a specific location for a minimum of 100 ms or four video frames and within 3° visual angle (or less) (Vickers, 1996; Williams et al., 1999). Mean number of fixations and number of areas of interest (AOIs) fixated upon per trial were determined.

#### Gaze Distribution

**FIGURE 1:**
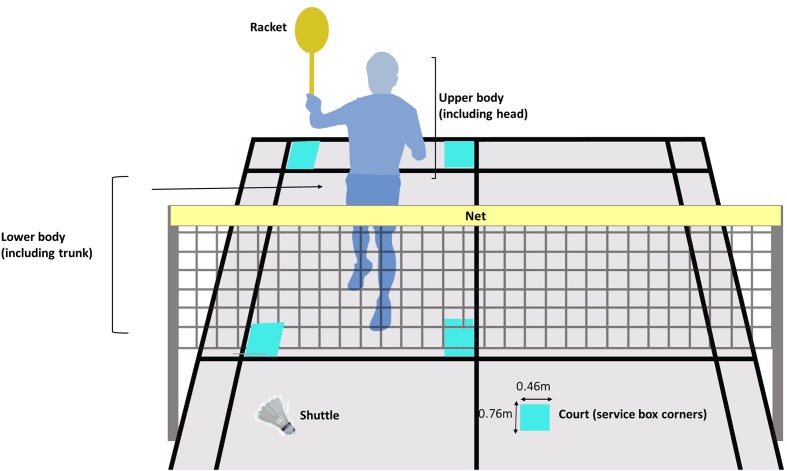
Areas of interest (AOI) identified in the current study (*n* = 6).

Gaze distribution was determined by calculating the total frequency count of data frames participants spent fixating on various locations in the environment when performing a badminton serve. Six AOIs were coded for analysis: (i) the upper body which includes the head, (ii) lower body, (iii) corners of service court (76 cm × 46 cm), (iv) opponent’s racket, (v) top portion of the net, and (vi) shuttle (refer to **Figure 1**). If the point of gaze was not in any of these areas, it was coded as “unclassified.” In addition, individual differences in gaze distribution in terms of percentage viewing time for each AOI were also determined by dividing the fixation duration of each AOI over the total fixation duration for each trial before averaging it over the total number of trials for each participant.

#### Quiet Eye Duration and Location

Quiet eye was operationally defined as the final fixation on a specific target for a minimum of four frames prior to the forward arm movement before racket-shuttle contact (Vickers, 1996). The frequency of occurrence of each AOI as the final fixation was also recorded to provide an indication of the AOI’s relative importance for the execution of the serve.

### Data Analysis

Visual behaviors of the participants during the serve test were analyzed frame-by-frame using the ASL Results Plus software. Serve type for each trial was recorded but as participants were not instructed on which type of serve to use, this factor was not considered in any of the statistical procedures. For each variable measured, the mean values were calculated and used for subsequent analyses. Statistical analysis was undertaken using SPSS version (22.0). An initial analysis of the data revealed that the assumption for normality had not been met, with the exception of the data relating to the shuttle and unclassified area. The normality of the dependent variables was assessed using the Shapiro–Wilk test as well as normality plots and estimates of skewness and kurtosis. Therefore, the non-normal variables were subjected to a log transformation to normalize the data before analysis (Wood and Wilson, 2010).

A non-parametric test was performed to compare mean trial length between the two groups of participants. Gaze distribution was analyzed using a 2 (skill) × 7 (AOI) Chi-square test. To determine the between-group differences for each AOI, 95% confidence intervals (CIs) were calculated. Differences between the two groups for number of fixations, number of fixation locations, QE duration and location, were analyzed using separate one-way between subjects ANOVAs. All relevant interactions and main effects were followed up using Fisher’s LSD *t*-tests, and effect sizes were calculated using partial eta squared ($\eta_{p}^{2}$). The significance level was set at *p* < 0.05. Note that although the inferential statistics were performed using transformed data, the means and standard deviations (SDs) that are reported in the text, tables and figures reflect the observed (not transformed) values.

## Results

Of the 720 trials, data from four trials (0.6%) were lost due to technical difficulties encountered with the eye-tracking equipment (Skilled: 1; Less skilled: 3). Out of the 716 remaining trials, 54 (7.5%) had no recorded fixations (Skilled: 21; Less skilled: 33). Thus, a total of 662 trials of the original 720 trials (91.9%) were analyzed.

### Trial Length

A Mann–Whitney test indicated that mean trial length approached significance, *U* = 38.5, *p* = 0.05. The skilled players took an average 2571 ± 1100 ms to serve as compared to their less skilled counterparts who took 1986 ± 360 ms.

### Fixation Count and Location

There were significantly greater number of fixations observed for the skilled as compared to the less skilled players, *F*(1,23) = 4.569, *p* = 0.044, $\eta_{p}^{2}$ = 0.172. The skilled players had an average of 2.4 ± 1.2 number of fixations per trial as compared to 1.6 ± 0.4 fixations in the less skilled players. In addition, there was a significant effect for the number of fixation locations per trial, *F*(1,23) = 6.186, *p* = 0.021, $\eta_{p}^{2}$ = 0.219. The skilled players fixated on significantly greater number of locations per trial than the less skilled (Skilled: 2.0 ± 0.7 vs. Less skilled: 1.5 ± 0.3).

### Gaze Distribution

**FIGURE 2:**
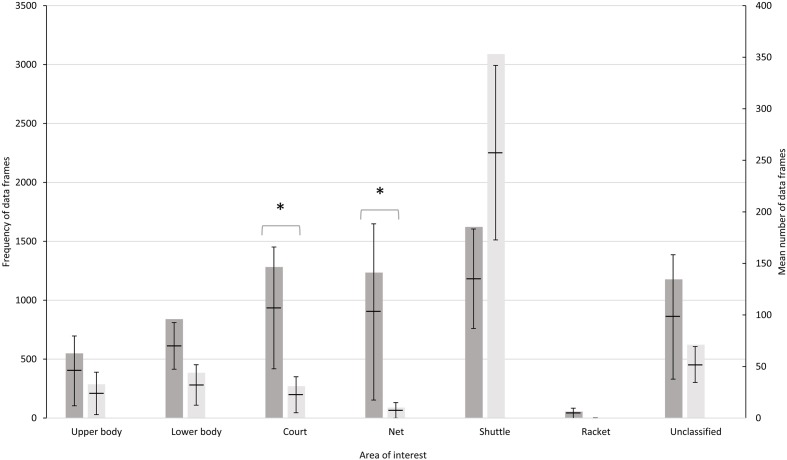
Frequency of data frames, mean (black line) and 95% confidence borders for each AOI for 662 trials for skilled (shaded) and less skilled participants. The asterisks represent significant differences (*p* < 0.05).

The number of data frames for each AOI (*n* = 6) of each skill group is presented in **Figure 2**. There was a significant association between the distribution of gaze and skill level, χ^2^(6) = 2300.90, *p* < 0.001, Cramer’s V = 0.45. Skilled players spent a significantly greater amount of time looking at the court (Skilled: 1282 frames vs. Less skilled: 271 frames) and net (Skilled: 1235 frames vs. Less skilled: 89 frames) than the less skilled. It was noted that there was no significant difference in the frequency count of the “unclassified” regions which included areas such as the participant’s own service court areas, his/her shoes, regions outside the court area for instance.

**FIGURE 3:**
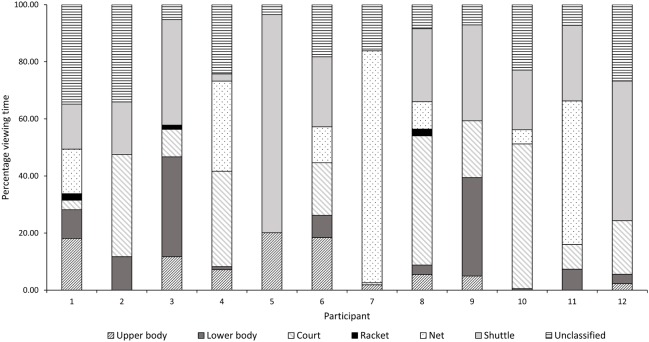
Mean and standard deviation of time spent viewing each AOI by individual participants (skilled) across conditions as a percentage of total viewing time.

**FIGURE 4:**
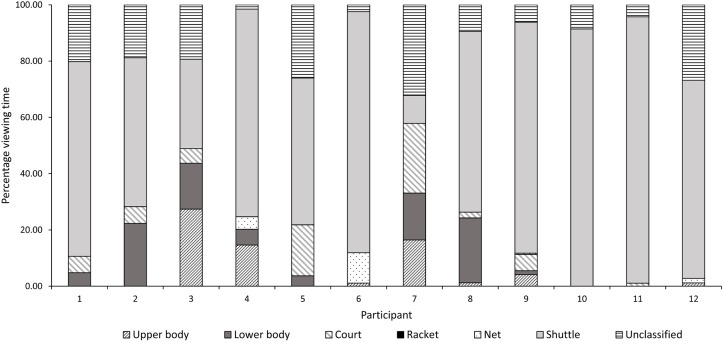
Mean and standard deviation of time spent viewing each AOI by individual participants (less skilled) across conditions as a percentage of total viewing time.

A closer examination of the areas fixated upon revealed considerable individual differences especially amongst the skilled players. **Figures 3**, **4** illustrate the individual data in terms of percentage viewing time for the skilled and less skilled players, respectively. The visual patterns for the less skilled players as seen from the mean data, shows the shuttle location dominating viewing time in 10/12 players (∼83%). Greater variability in visual strategies was observed for the skilled players. For instance, Participants 1 and 7 from the skilled group exhibited entirely different visual patterns. Participant 7 predominantly fixated on the net whereas Participant 1, looked at all locations. Interestingly, the distribution of viewing times for Participant 8 of the skilled group bears close resemblance to that of the less-skilled players. For the court location, 8/12 skilled players (75%) spent an appreciable percentage of viewing time on this area compared with only 2/12 (∼17%) of less skilled players.

### Quiet Eye Duration and Location

**FIGURE 5:**
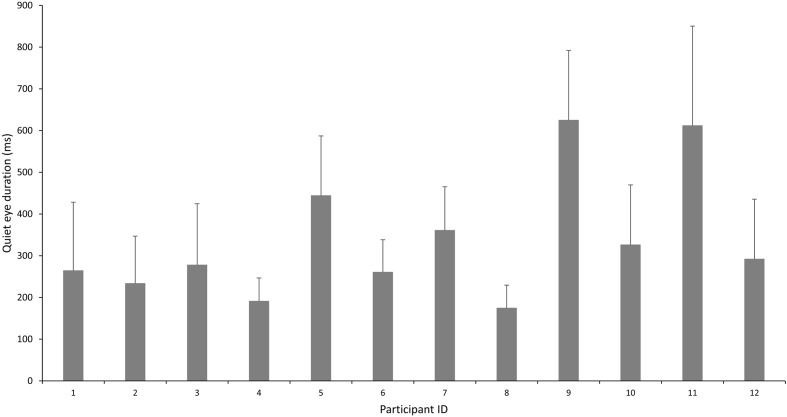
Inter-individual variation in quiet eye duration (ms) amongst the less skilled participants.

The less skilled players displayed longer QE durations (Less skilled: 338.23 ± 149.41 ms vs. Skilled: 291.92 ± 78.69 ms). Individual QE durations for the less skilled are presented in **Figure 5**. With regards to QE location, a significant main effect was found, *F*(6,132) = 30.135, *p* < 0.001, $\eta_{p}^{2}$ = 0.578. This was accompanied by a significant interaction effect, *F*(6,132) = 5.328, *p* = 0.014, $\eta_{p}^{2}$ = 0.195 between QE location and skill level. *Post hoc* tests revealed skill-related differences in the percentage of occurrence of the court, net and shuttle as the final fixation location. There was a significantly larger percentage of occurrence of the court and net as the final fixation amongst the skilled players (Court: 16.6 ± 20.7%; Net: 19.0 ± 25.2%) as compared to the less skilled (Court: 2.9 ± 5.7%; Net: 0.3 ± 1.0%). There was however, significantly greater percentage of occurrence of the shuttle for the less skilled (Skilled: 80.4 ± 19.4% vs. Less skilled: 42.6 ± 34.7%).

## Discussion

In this study, visual behavior of players (server) during the badminton serve was examined. The serve is a key opportunity in badminton with each rally beginning with the serve and when executed accurately, this presents the opportunity for the server to implement their offensive strategy, for example, a smash or drop shot (Tong and Hong, 2000). Apart from being able to accurately deliver the shuttle to the desired spot, it is advantageous for one to minimize any visual information present to the opponent, taking him by surprise. This study investigated if players of different skill levels would exhibit different visual behaviors as they served. Findings indicate key differences in visual search strategies as a function of skill. Skilled players had more fixations on a greater number of locations per trial as compared to their less skilled counterparts. Moreover, there is a distinct difference in how skilled players had higher frequency counts on their target – court and net – as compared with the less skilled who fixated predominantly on the object for striking – the shuttle. Below, we discuss the reasons for such differences and the implications in terms of movement control and future practice interventions.

It was observed that the skilled players took a longer period of time before serving as compared to their less skilled counterparts (2571 ms vs. 1986 ms). In badminton, the server has two aims, first to ensure that the shuttle is delivered to the appropriate service court and at the desired spot and secondly, minimize the chances of a successful offensive shot by the opponent (Grice, 2007). While the greater number of fixations and AOI fixated upon provides support for our hypothesis, these results contrast previous work (see Mann et al., 2007 for a review) where visual behavior of higher skilled players were characterized by fewer fixations. While no points were awarded for the serving task in this study, it is likely that the skilled players were accustomed to the importance of a serve and were thus, more deliberate in their actions. This could have possibly led to the emergence of a longer and more diverse fixation pattern.

Our findings also contradict badminton anticipatory studies where no between-group differences for the number of fixations were observed (Abernethy and Russell, 1987b; Alder et al., 2014). Here, it is important that we consider the type of task as well as the presence of any temporal limits. In badminton, there is no rule to stipulate that a player must serve within a specific time-period. Contrastingly, players have approximately 0.1 s to react to a shot from their opponents (Bańkosz et al., 2013). Therefore, when a movement response has to be made within a short period of time (e.g., returning a shot from the opponent), one possibility is that higher skilled individuals would employ a more efficient visual pattern by relying on their knowledge base to extract the most relevant informational constraints, resulting in a fewer number of fixations recorded. This, however, may not apply for self-paced tasks where there is ample preparatory time before performing the movement. Alternatively, the number of fixations may not differ as a function of skill but fixation location, duration as well as information utilization differentiates individuals of different skill levels.

Moreover, the visual pattern observed in the present study may have been a result of an intentional attempt to manipulate the anticipatory informational constraints by looking at multiple locations to prevent their intended serve direction and/or location from being anticipated (Jackson et al., 2006). The representativeness of the current task (with an opponent) may have allowed the servers to utilize the affordances typically observed in a real game context. This is confirmed by skilled Player 8 who shared anecdotally that he would intentionally look at the front corners of the service court but serve a long one to the back or vice versa, depending on the opponent’s position.

The categorization of “court” in this study referred to the corners of the service court. Serving to the extreme corners of the service court forces the opponent further away from the “base” or central position of the court (Downey, 1982). Being the best position from which to defend your court, this “base” position allows the player to reach all four corners of the court equally well (Wadood, 2014). Secondly, sending the shuttle low over the net makes it hard for the opponent to return well unless he is positioned well to the front of the court with racket above the top of the net waiting to “attack” the shot (Mack, 2016). This forces the opponent to lift the shuttle upward, allowing the server the opportunity to implement his attack with a smash or drop shot for example. This was observed by Tong and Hong (2000) who found that 7 out of the 11 elite singles players analyzed, preferred to use the short serve to facilitate their offensive strategy as evidenced by the smash shot being the most popular shot played to win the rally. As such, the chance of winning the rally is greatly increased if the serve is executed and placed well. The serve is thus crucial in determining the success of subsequent shots in the rally. Skilled players have been shown to demonstrate greater ability to assess their opponent’s position when serving to determine the space which makes the return of their serves most difficult (Del Villar et al., 2007). As a receiver, depending on what the player perceives to be the main threat, this would affect the standing position. For instance, to guard against a long serve, the receiver would take up a slightly backward attacking stance during service (Downey, 1982). Moreover, the receiver can intentionally position himself to “encourage” the opponent to execute certain serves to his advantage. These subtle but important adjustments to the receiver’s position may have an impact on the choice of serve delivered. Such informational constraints, however, are probably not salient to the less skilled players where the temporal-spatial constraint to hit the shuttle is of more significance than the accuracy of the serve. These less skilled players may be more focused on getting the shuttle across the net as this is a more important constraint (height clearance rather than accuracy). In this study, this is reflected in both the court and net being among the least important fixation locations for the less skilled players (Court: 271 out of 4744 data frames – 5.71%; Net: 89 out of 4744 data frames – 1.88%).

Irrespective of skill level, the shuttle was the AOI that was fixated the most during the trial (see **Figure 2**). Moreover, results from this study also indicated that the shuttle served as an important final point of fixation prior to the serve. The shuttle made up the highest percentage of occurrence, at 24.0 and 65.1%, respectively for the skilled and less skilled players. Firstly, the dependence on the shuttle as a source of visual information can be explained in terms of action guidance. In order to ensure accurate contact between the racket and shuttle, the eyes steer the individual toward the shuttle, providing the necessary directional guidance to the motor system (Land, 2009). This is typical in similar aiming tasks such where an object must be accurately struck toward a target (e.g., golf putting). This, however, may be more applicable for the less skilled players. Alternatively, considering the automaticity of the serving action for the skilled players, fixating on the shuttle could be another strategy to prevent the opponents from anticipating the serve direction and placement. While there is no pressure to perform the serve in the shortest possible time, the lack of a temporal constraint guarantees that at some point, the opponent will be provided with invariant visual information that is highly predictive of the forthcoming serve (Farrow and Abernethy, 2015). As such, the skilled server could be fixating on the shuttle to minimize the provision of informational constraints to the receiver for any prediction on where the serve may be targeting.

Surprisingly, we were unable to replicate significant differences in QE durations. Furthermore, QE duration was longer for the less skilled as compared to the skilled players. This could have resulted from the lack of familiarity and perceived confidence (lack of) in performing the serve as compared to the skilled players. This translates to a longer aiming period prior to performing the serve. It is important to note that especially for less skilled individuals, a longer QE does is not necessarily useful. Horn et al. (2012) found no correlation between QE duration and accuracy in a dart aiming task performed by less skilled individuals. This suggests that rather than being a causal factor in successful aiming, longer QE needs to be coupled with other factors such as the individual’s technical skill. Alternatively, the presence of large individual variability might have also affected the results. For instance, less skilled Player 9 and 11 exhibited overly exaggerated QE durations (>600 ms) as compared to all other participants in this study. Similar variability between individuals even of similar skill levels were reported in QE characteristics within levels of expertise in a self-paced aiming bowling task (Chia et al., 2016). Hence, the failure to find significant skill-related differences in this study may have implications on the importance of QE in similar self-paced aiming tasks, especially those constrained by the presence of an opponent (e.g., tennis serve). Caution must be taken when attempting to transfer QE findings from other aiming tasks.

Interestingly, even amongst the skilled players, distinct differences in visual behavior were observed. Some players fixated upon minimal number of locations (e.g., skilled Player 7 fixated predominantly on the net) whereas others took in information from every location identified (e.g., skilled Player 1). As such, while significant skill-related differences in the number of fixations and locations were found, it is possible for variability to be present in visual patterns even within skill levels (Dicks et al., 2010). While one may argue that these variabilities arise because of different serving actions, both skilled players in this case adopted the same short serve type. This gives rise to the possibility that serve type alone may not explain why players look toward certain cues during the badminton serve. To ensure that the task was as representative as possible, serve type was not controlled for in this study; there were insufficient number of each serve type for further analysis. Results from the study may, however, be limited by the possible expertise differences within the skilled group (college vs. national player) despite controlling for the number of years of competitive experience.

In summary, these preliminary findings showed for the first time, skill-related differences in visual behavior during a badminton serve task. It is apparent that the nature of the task and the visual informational constraints presented, largely influenced the underlying visual behavior of the players. This proposition highlights the critical role that interaction among constraints play in the emergence of different movement behaviors (see Chow et al., 2016). Skilled players used a search strategy involving a greater number of fixations to more locations of the visual display than less skilled players. It cannot be determined from this study whether these fixations were part of one’s aiming process or an attempt to manipulate the visual information presented to the opponent. Inter-individual comparisons of visual strategies also highlighted the possibility that serving strategies may be individual specific, especially amongst the skilled players. Moreover, based on this current study, it is not clear which serving strategy is most effective. Considering the inter-individual variability and possible deception involved in the badminton serve, a shift from group to individual analyses is recommended for future studies (Dicks et al., 2010). It would also be interesting to examine the differences in these visual strategies with and without an opponent, coupled with serve performance to evaluate the effectiveness of these strategies. This is important especially from a coaching and/or training perspective so coaches do not instruct players to fixate on less meaningful cues. Finally, there is a need to extend this research to look at the doubles discipline where the serve may be even more important.

## Ethics Statement

This study was carried out in accordance with the recommendations of Nanyang Technological University (NTU) Institutional Review Board with written informed consent from all subjects. All subjects gave written informed consent in accordance with the Declaration of Helsinki. The protocol was approved by the NTU Institutional Review Board.

## Author Contributions

All authors contributed extensively to the work presented in the paper. JSC, SB, and JYC obtained funding and contributed to the study design. JSC collected the data and performed the analyses. All authors contributed to the data interpretation, drafting of the manuscript as well as the final approval of the work to be published.

## Conflict of Interest Statement

The authors declare that the research was conducted in the absence of any commercial or financial relationships that could be construed as a potential conflict of interest.
